# Camphor and Chloroform Mixture

**Published:** 1849-05

**Authors:** 


*Camphor and Chloroform Mixture.* By T. & H. Smith. (*Monthly Jour. & Retrosp. of the Medical Sciences*, Nov. 1848.)—There is great difficulty, or rather an utter impossibility of administering camphor in a state of solution in doses of sufficient potency in some cases. The form of pill, the only mode of giving large doses of this medicine, is objectionable in many cases, and in others altogether inadmissable. The camphor being merely in a state of mechanical division, on being set free in the stomach, from its extreme lightness quickly separates and floats about, thus producing in many cases much local irritation in that organ, instead of soothing or arousing the general system.

Messrs. T. & H. Smith, Chemists of Edinburgh, give a formula for exhibiting camphor in doses of almost any amount of strength — certainly as large as any case can require — and that in a state of perfect solution; thereby allowing of a nice adaptation of the dose to the circumstances of each case.

The formula is as follows:— Three drachms of solid camphor are dissolved in one fluid drachm of chloroform. This is, perhaps, one of the most remarkable cases of solution the whole range of chemistry presents to us. The solution is most *rapid and complete*, and the bulk of the liquid is now increased from one to fully four liquid drachms. This solution rubbed up with the *yolk* of one fresh egg, may be formed into an extremely elegant emulsion by the addition of water, without the slightest separation of the camphor or chloroform; in fact, no separation of any kind takes place. If to the proportions given above as much water be added as to make a four ounce mixture, each tea-spoonful of the mixture when formed will contain about five and a half grains of camphor, and about two minims of chloroform. The capability of the formula being varied, either the camphor or chloroform may constitute the predominating ingredient, must be quite obvious. This mixture can be administered in any ordinary vehicle, such as water, without the occurrence of any separation; indeed, the mixture is as readily and completely effected as cream with tea or coffee. We have tried the effect of several medical substances on the mixture. With none of them has any separation been caused.

